# Impact of a robotic approach on hypoattenuated area formation leading to postoperative pancreatic fistula in patients after pancreatoduodenectomy

**DOI:** 10.1007/s00464-025-11635-2

**Published:** 2025-03-04

**Authors:** Yoshito Tomimaru, Shogo Kobayashi, Kazuki Sasaki, Shinichiro Hasegawa, Daisaku Yamada, Hirofumi Akita, Takehiro Noda, Hidenori Takahashi, Hiroki Imamura, Yuichiro Doki, Hidetoshi Eguchi

**Affiliations:** Department of Gastroenterological Surgery, Graduate School of Medicine, Osaka University, 2-2 Yamadaoka E-2, Suita, Osaka 565-0871 Japan

**Keywords:** Hypoattenuated area, Pancreaticoduodenectomy, Pancreatic fistula, Pancreatojejunostomy, Robotic pancreaticoduodenectomy

## Abstract

**Background:**

Hypoattenuated area (HA) formation at the pancreatojejunostomy (PJ) site on contrast-enhanced computed tomography (CE-CT) is significantly associated with clinically relevant postoperative pancreatic fistula (CR-POPF) after open pancreaticoduodenectomy (PD) (O-PD). Here, we evaluated the impact of HA formation in robotic PD (R-PD) and surgical factors predictive of HA formation.

**Methods:**

The study retrospectively analyzed 66 patients who underwent either O-PD or R-PD and exhibited a drain amylase level exceeding three times the upper limit of normal range, with CE-CT assessment performed on postoperative days 3–14. Patients were divided into two groups, with evident HA (≥ 5 mm) (E-HA) and subtle HA (< 5 mm) (S-HA), and their data were analyzed by multivariate and propensity-score matching analyses.

**Results:**

Among the patients, 24 (36.3%) exhibited E-HA and 42 (63.7%) S-HA. The percentages of R-PD and CR-POPF in E-HA group were significantly lower and higher, respectively, than S-HA group (R-PD: 29.2% vs 54.8%, *p* = 0.0446; CR-POPF: 70.8% vs 4.8%, *p* < 0.0001). Multivariate analysis revealed the surgical approach as a significant factor associated with E-HA formation (odds ratio: 0.26; *p* = 0.0223). Propensity-score matching analysis revealed significantly fewer patients with E-HA formation and CR-POPF in R-PD group than O-PD group (E-HA: 14.3% vs 64.3%, *p* = 0.0068; CR-POPF: 14.3% vs 57.1%, *p* = 0.0180).

**Conclusion:**

The impact of HA formation in predicting CR-POPF was confirmed in the patients undergoing PD, including O-PD and R-PD. Furthermore, the data suggest that R-PD, compared with O-PD, significantly decreased the incidence of E-HA formation, indicating an advantage of R-PD over O-PD in reducing CR-POPF via HA formation.

Pancreaticoduodenectomy (PD) has been considered the surgical gold standard for both benign and malignant tumors located in the periampullary region, including the pancreatic head, despite its complexity [1–3]. Once performed only via an open approach, following the introduction of minimally invasive surgery, laparoscopic PD was successfully performed by Gagner et al. in 1994 [4]. Subsequently, with the spread of robotic surgery, robotic PD (R-PD) was first reported by Giulianotti et al. in 2003 [5], and since then it has spread rapidly. Concomitantly, some studies have highlighted the potential advantages of R-PD over open PD (O-PD) [6–11]. For example, a multicenter retrospective study reported significantly reduced intraoperative blood loss and postoperative hospital stay in patients undergoing R-PD when compared with O-PD [6]. However, a recent randomized controlled trial exhibited significantly higher incidence of pancreas-specific complications and delayed gastric emptying in the R-PD group than in the O-PD group [12]. Thus, the advantage of R-PD over O-PD remains inconclusive.

Postoperative pancreatic fistula (POPF) remains one of the most common complications after pancreatectomy, including PD [6, 13–15]. The International Study Group on Pancreatic Surgery (ISGPS) categorized POPF into three categories based on its severity: biochemical leak (BL), which is no longer considered a fistula, and grades B and C, which are both recognized as clinically relevant POPF (CR-POPF) [16]. This categorization highlights the clinical importance of differentiating between CR-POPF and BL POPF, but the differentiation remains challenging. Recently, we reported a significant association between the incidence of CR-POPF and the formation of a hypoattenuated area (HA), which is sometimes identified at the pancreatojejunostomy (PJ) site on contrast-enhanced computed tomography (CT) (CE-CT) in patients who have received PD [17]. In that report, we also investigated the diagnostic value of HA formation for CR-POPF, and the radiological evaluations of HA suggested that reduced blood supply in the remnant pancreas might lead to HA formation. Thus, the study raised the possibility that patients experiencing BL POPF may benefit from CE-CT to predict CR-POPF. However, the study included only patients who underwent O-PD, as it was conducted before the prevalence of R-PD in our institution, implying that the impact remains unclear in patients receiving R-PD. Furthermore, there have been no investigations regarding factors predictive of HA formation. Given the clinical importance of HA formation in predicting CR-POPF progression, investigating factors predictive of HA formation would be useful in helping predict CR-POPF via HA formation. Here, on the basis of this background, we first verified the impact of HA formation on CR-POPF in consecutive patients who underwent PD, including both O-PD and R-PD. The verification also suggested a decreased risk of HA formation with the robotic approach compared with that of the open approach. Based on the suggestion, we also investigated factors predictive of HA formation with a focus on the surgical approach. Focusing on the surgical approach would be useful also in the current situation where the advantage of R-PD over O-PD in pancreatic surgery remains inconclusive.

## Materials and methods

### Patients

We retrospectively reviewed the cases of 171 consecutive patients who underwent PD with PJ reconstruction at the Department of Gastroenterological Surgery, Osaka University Hospital between January 2021 and March 2024. Of the 171 patients, 66 patients met the diagnostic criterion of BL, defined as a drain amylase level exceeding three times the upper limit of the institutional normal range (> 459 U/L), and were subjected to CE-CT assessment on postoperative days (PODs) 3–14 [16]. The 66 patients were included in this study.

After an extensive dialogue with the Institutional Ethics Review Committee of Osaka University Hospital, patient consent for participation was obtained through an opt-out method. This study was approved by the Institutional Ethics Review Committee (Certificate Number 22096).

### Surgical procedure and postoperative management

All included patients underwent subtotal stomach-preserving PD. After resection of the pancreatic head, reconstruction was performed in the following order: pancreas, bile duct, and stomach. The PJ anastomosis was performed using 3–0 nonabsorbable monofilaments with the modified Blumgart method [18] following duct-to-mucosa anastomosis using 5–0 absorbable monofilaments. In each patient, a pancreatic stent tube (PST) was placed to internally or externally drain the pancreatic juice. The PD procedure and the surgeons were the same for both O-PD and R-PD. R-PD was performed using the da Vinci Xi surgical system (Intuitive Surgical, Inc., Sunnyvale, CA, USA). Only O-PD was indicated for patients who were preoperatively planned to have combined resection of major vessels and other organs during the operation. For the other patients, R-PD was indicated if the robotic surgical system was available; otherwise, O-PD was indicated.

POPF was graded according to the definition proposed by the ISGPS [16]. Other postoperative complications were defined; complications were defined as Clavien–Dindo classification grade ≥ 3 [19]. All patients underwent the same postoperative management in accordance with our institutional policy regardless of whether the surgical approach was O-PD or R-PD [17, 20]. Briefly, surgical drains were placed under the hepatoduodenal ligament and ventral and dorsal sides of the PJ anastomosis. The drain amylase level was measured on PODs 1 and 3 and thereafter at intervals of 2–3 days until drain removal. Octreotide was administered when the amylase concentration in the drainage fluid was > 5000 U/L. Patients experiencing BL were further examined by CT scan for any signs of CR-POPF development; otherwise, the abdominal drains were subsequently removed. In patients that developed CR-POPF, the intra-abdominal drainage tube was changed every 1–2 weeks, and the drainage tube was removed when the patients were asymptomatic and imaging modalities confirmed the disappearance of the intra-abdominal cavity independently of appearance or amylase concentration in the drainage fluid through the tubes.

### Definition of HA

**Fig. 1:**
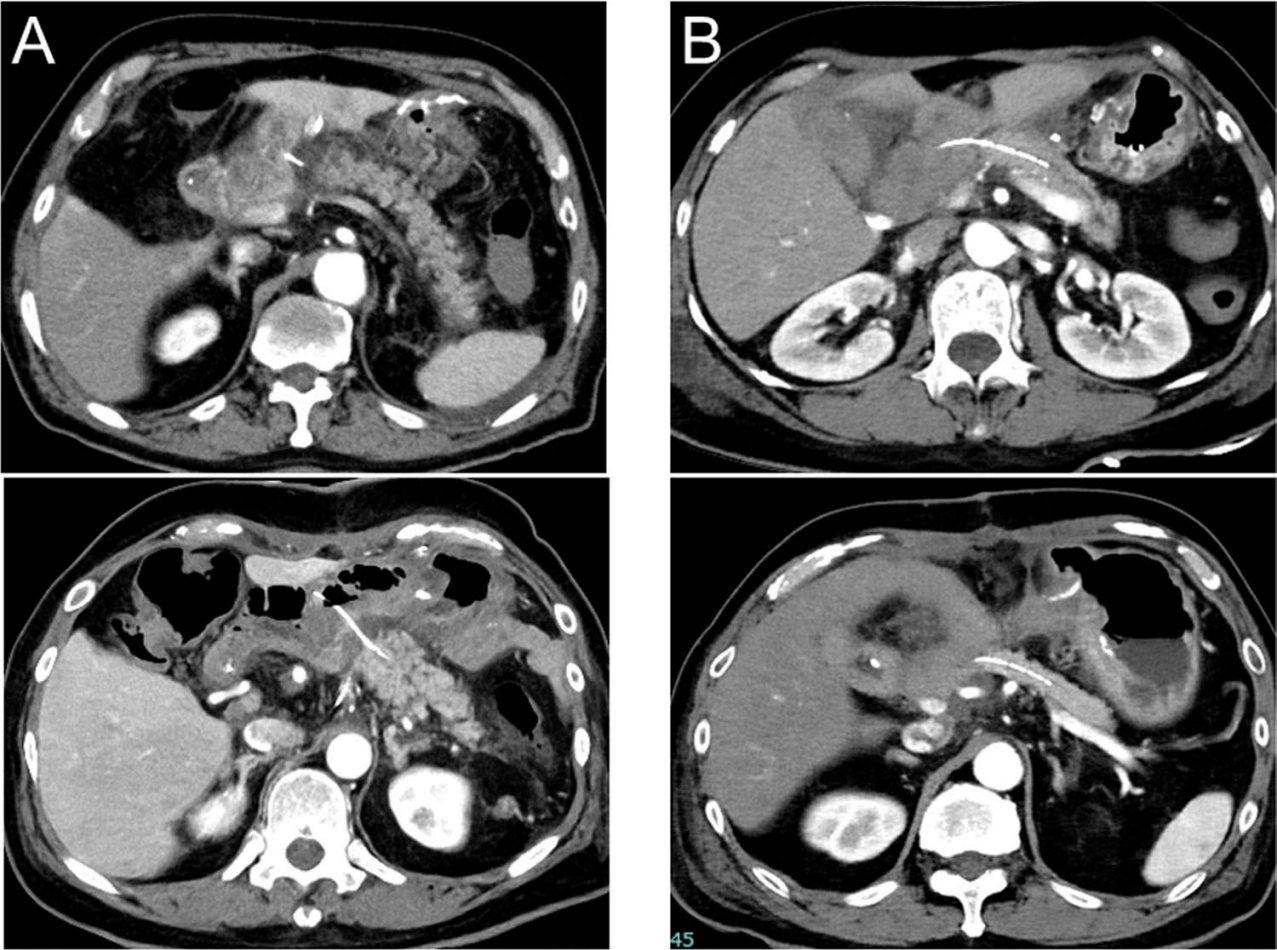
Representative images of E-HA and S-HA. The pictures show representative image of E-HA (**A**) and S-HA (**B**) on CE-CT. CE-CT, contrast-enhanced computed tomography; E-HA, evident hypoattenuated area; S-HA, subtle hypoattenuated area

HA was defined as previously reported [17]. Briefly, HA was defined as a low-density area at the PJ site. The presence or absence of HA was evaluated on CE-CT. When present, the HA length was measured as the distance from the jejunal wall to the well-enhanced remnant pancreas along the PST. When HA was absent on CE-CT, the HA length was recorded as zero. Based on the HA measurements, the patients were categorized into two groups: an evident HA (E-HA) group including patients with HA ≥ 5 mm, and a subtle HA (S-HA) group including patients with HA < 5 mm. Representative CT images of E-HA and S-HA are shown in Fig. 1.

### Statistical analysis

Measured data were described as mean values ± standard deviations for continuous variables, and as numbers for categorical variables. Differences between groups were assessed with the chi-square test, Fisher’s exact test, or the Mann–Whitney U test. Logistic regression analysis was performed to identify factors associated with a targeted event. Propensity-score matching (PSM) analysis was conducted to compare the groups, adjusting for confounders from a different perspective from that of the multivariate analysis. Specifically, we used the 1:1 nearest-neighbor matching method with a caliper width of 0.20 for the standardized difference of logit-transformed propensity scores. The covariates included body mass index (BMI), main pancreatic duct (MPD) diameter, neoadjuvant therapy, and tumor location. These factors were selected because they exhibited significant differences between the two groups. Statistical analyses were performed with JMP Pro 14 software (SAS Institute Inc., Cary, NC). *P* values < 0.05 were considered statistically significant.

## Results

### Comparison of clinical characteristics of patients with E-HA vs S-HA

Among the 66 patients, E-HA was identified in 24 patients (E-HA group: 36.3%), and S-HA in the remaining 42 patients (S-HA group: 63.7%). The incidence of E-HA was notably lower than in our previous study (43.8%) [17]. The clinical characteristics of the 24 patients in the E-HA group were compared with those in the S-HA group (Table 1). The percentage of R-PD was significantly lower in the E-HA group than in the S-HA group (29.2% vs 54.8%; *p* = 0.0446), while the other preoperative or intraoperative factors did not differ significantly between the two groups. There was no significant difference in the interval from the surgery to CT scan between the E-HA group and the S-HA group (7 ± 3 days vs 7 ± 3 days; *p* = 0.3564). In terms of postoperative factors, the drain amylase level on POD3 was higher in the E-HA group than in the S-HA group, but this difference was not statistically significant. The incidence of POPF was significantly higher in the E-HA group than in the S-HA group (70.8% vs 4.8%; *p* < 0.0001), while there was no significant difference in the incidence of the other complication rate (0% vs 7.1%; *p* = 0.2951). The significant association between HA status and POPF confirmed the findings of our previous study [17]. Before investigating factors associated with HA status, we analyzed factors associated with CR-POPF (Table 2). Univariate and multivariate analyses suggested that HA status was an independent factor significantly associated with CR-POPF; the sensitivity and specificity of E-HA for predicting CR-POPF were 89.4% and 85.1%, respectively. These findings were consistent with the results of our previous study [17].

**Table 1:** Clinical characteristics of patients in the S-HA and E-HA groups

Factors	S-HA group (*n* = 42)	E-HA group (*n* = 24)	*P* value
Preoperative factors			
Age (years)	68 ± 14	66 ± 9	0.5985
Sex			0.6404
Male (%)	22 (52.4%)	14 (58.3%)	
Female (%)	20 (47.6%)	10 (41.7%)	
BMI (kg/m^2^)	22.1 ± 3.1	23.4 ± 2.4	0.0901
MPD diameter (mm)	2.6 ± 1.9	2.6 ± 2.3	0.9499
Pancreatic thickness (mm)	11.3 ± 2.5	11.5 ± 2.6	0.7019
Pancreatic texture			0.5301
Soft	40 (95.2%)	24 (100%)	
Hard	2 (4.8%)	0 (0%)	
Neoadjuvant therapy			0.2776
−	30 (71.4%)	14 (58.3%)	
+	12 (28.6%)	10 (41.7%)	
Tumor location			0.5332
Pancreas	27 (28.6%)	17 (16.7%)	
Bile duct	12 (66.7%)	4 (70.8%)	
Duodenum	1 (2.4%)	2 (8.3%)	
Others	2 (2.4%)	1 (4.2%)	
Intraoperative factors			
Approach			0.0446
O-PD	19 (45.2%)	17 (70.8%)	
R-PD	23 (54.8%)	7 (29.2%)	
Operation time (min)	512 ± 118	507 ± 127	0.8777
Intraoperative blood loss (mL)	328 ± 374	463 ± 542	0.2375
Intraoperative transfusion			0.5484
−	41 (97.6%)	22 (91.7%)	
+	1 (2.4%)	2 (8.3%)	
Postoperative factors			
Drain amylase on POD 3 (U/L)	8360 ± 16,795	18,286 ± 36,821	0.1383
Postoperative complication			
CR-POPF			< 0.0001
−	40 (95.2%)	7 (29.2%)	
+	2 (4.8%)	17 (70.8%)	
Other complications			0.2951
−	39 (92.9%)	24 (100%)	
+	3 (7.1%)	0 (0%)	

*BMI* body mass index, *CR-POPF* clinically relevant postoperative pancreatic fistula, *E-HA* evident hypoattenuated area, *MPD* main pancreatic duct, *O-PD* open pancreaticoduodenectomy, *POD* postoperative day, *R-PD* robotic pancreaticoduodenectomy, *S-HA* subtle hypoattenuated area

**Table 2:** Univariate and multivariate analysis for CR-POPF

Factor	Univariate	Multivariate		
	*P* value	OR	95% CI	*P* value
Preoperative factors				
Age (≥ 70 years)	0.3736			
Sex (Male)	0.4162			
BMI (≥ 22.8 kg/m^2^)	0.7858			
MPD diameter (≥ 3 mm)	0.7232			
Pancreatic thickness (≥ 12 mm)	0.7858			
Pancreatic texture (soft)	0.7009			
Neoadjuvant therapy ( +)	0.8476			
Tumor location (pancreas)	0.9811			
Intraoperative factors				
Approach (R-PD)	0.7284			
Operation time (≥ 520 min)	0.7858			
Intraoperative blood loss (≥ 280 mL)	0.4162			
Intraoperative transfusion ( +)	0.9768			
Postoperative factors				
Drain amylase on POD3 (≥ 4500 U/L)	0.0736	0.495	0.13–2.73	0.4954
HA (E-HA)	< 0.0001	43.48	8.20–250.00	< 0.0001
Other complications ( +)	0.9768			

*BMI* body mass index, *CI* confidence interval, *CR-POPF* clinically relevant postoperative pancreatic fistula, *E-HA* evident hypoattenuated area, *HA* hypoattenuated area, *MPD* main pancreatic duct, *OR* odds ratio, *POD* postoperative day, *R-PD* robotic pancreaticoduodenectomy

### Factors associated with E-HA status

The above results suggested a possible significant correlation between the PD surgical approach and E-HA status, which was in turn significantly associated with CR-POPF. Based on this possibility, we performed univariate and multivariate analyses to investigate whether the surgical approach was significantly associated with E-HA status (Table 3). The univariate analysis identified two factors, the surgical approach (R-PD/O-PD) and drain amylase on POD3, as significantly associated with E-HA (*p* = 0.0481, *p* = 0.0438, respectively). In the multivariate analysis using these two factors, the surgical approach (R-PD/O-PD) remained significantly associated with E-HA status (odds ratio: 0.26, 95% confidence interval: 0.08–0.83; *p* = 0.0223). Thus, the statistical analyses suggested a significant association between the surgical approach and E-HA status.

**Table 3:** Univariate and multivariate analysis of E-HA status

Factor	Univariate	Multivariate		
	*P* value	OR	95% CI	*P* value
Preoperative factors				
Age (≥ 70 years)	0.6406			
Sex (Male)	0.5931			
BMI (≥ 22.8 kg/m^2^)	0.6091			
MPD diameter (≥ 3 mm)	0.8452			
Pancreatic thickness (≥ 12 mm)	0.6901			
Pancreatic texture (soft)	0.9805			
Neoadjuvant therapy ( +)	0.2800			
Tumor location (pancreas)	0.5878			
Intraoperative factors				
Approach (R-PD)	0.0481	0.26	0.08–0.83	0.0223
Operation time (≥ 520 min)	> 0.9999			
Intraoperative blood loss (≥ 280 mL)	0.6290			
Intraoperative transfusion ( +)	0.2937			
Postoperative factors				
Drain amylase on POD3 (≥ 4500 U/L)	0.0438	3.87	1.23–12.11	0.0203
Other complications ( +)	0.9761			

*BMI* body mass index, *CI* confidence interval, *CR-POPF* clinically relevant postoperative pancreatic fistula, *E-HA* evident hypoattenuated area, *HA* hypoattenuated area, *MPD* main pancreatic duct, *OR* odds ratio, *POD* postoperative day, *R-PD* robotic pancreaticoduodenectomy

### Comparison between O-PD and R-PD

Based on the significant association between the surgical approach and E-HA status, patients who received O-PD and R-PD were compared in regard to perioperative factors. As shown in Table 4, some factors differed significantly between the two groups. Specifically, BMI was significantly higher in the R-PD group than in the O-PD group (23.4 ± 2.7 kg/m^2^ vs 21.9 ± 3.0 kg/m^2^; *p* = 0.0438). MPD diameter was significantly smaller in the R-PD group than in the O-PD group (2.0 ± 1.2 mm vs 3.1 ± 2.4 mm; *p* = 0.0218). The percentage of patients receiving neoadjuvant therapy was significantly lower in the R-PD group than in the O-PD group (10.0% vs 52.8%; *p* = 0.0002), and the distribution of tumor location differed significantly between the two groups (*p* = 0.0275). In terms of intraoperative factors, the R-PD group exhibited significantly longer operation times and less intraoperative blood loss (582 ± 1.2 min vs 450 ± 104 min; *p* < 0.0001) (192 ± 270 mL vs 531 ± 501 mL; *p* = 0.0015). No other preoperative or intraoperative factors differed significantly between the two groups. Regarding postoperative factors, drain amylase level on POD3 was significantly higher in the R-PD group than in the O-PD group (20,195 ± 36,693 U/L vs 51,144 ± 6518 min; *p* = 0.0182), and the percentage of patients with E-HA was significantly lower in the R-PD group than in the O-PD group (23.3% vs 47.2%; *p* = 0.0446). The incidence of E-HA in the O-PD group (47.2%) was similar to that observed in our previous study (43.8%) [17]. On the other hand, there was no significant difference in the incidence of CR-POPF or other complications.

**Table 4:** Clinical characteristics of patients in the O-PD and R-PD groups

Factors	O-PD group (*n* = 36)	R-PD group (*n* = 30)	*P* value
Preoperative factors			
Age (years)	66 ± 10	68 ± 15	0.5440
Sex			0.4166
Male (%)	18 (50.0%)	18 (60.0%)	
Female (%)	18 (50.0%)	12 (40.0%)	
BMI (kg/m^2^)	21.9 ± 3.0	23.4 ± 2.7	0.0438
MPD diameter (mm)	3.1 ± 2.4	2.0 ± 1.2	0.0218
Pancreatic thickness (mm)	11.1 ± 2.7	11.7 ± 2.3	0.3646
Pancreatic texture			0.5301
Soft	34 (94.4%)	30 (100%)	
Hard	2 (5.6%)	0 (0%)	
Neoadjuvant therapy			0.0002
−	17 (47.2%)	27 (90.0%)	
+	19 (52.8%)	3 (10.0%)	
Tumor location			0.0275
Pancreas	27 (28.6%)	17 (16.7%)	
Bile duct	4 (66.7%)	12 (70.8%)	
Duodenum	2 (2.4%)	1 (8.3%)	
Others	3 (2.4%)	0 (4.2%)	
Intraoperative factors			
Operation time (min)	450 ± 104	582 ± 97	< 0.0001
Intraoperative blood loss (mL)	531 ± 501	192 ± 270	0.0015
Intraoperative transfusion			> 0.9999
−	34 (94.4%)	29 (96.7%)	
+	2 (5.6%)	1 (3.3%)	
Postoperative factors			
Drain amylase on POD 3 (U/L)	5114 ± 6518	20,195 ± 36,693	0.0182
HA			0.0446
S-EA	19 (52.8%)	23 (76.7%)	
E-HA	17 (47.2%)	7 (23.3%)	
Postoperative complication			
CR-POPF			0.7283
−	25 (69.4%)	22 (73.3%)	
+	11 (30.6%)	8 (26.7%)	
Other complications			> 0.9999
−	34 (94.4%)	29 (96.7%)	
+	2 (5.6%)	1 (3.3%)	

*BMI* body mass index, *CR-POPF* clinically relevant postoperative pancreatic fistula, *E-HA* evident hypoattenuated area, *MPD* main pancreatic duct, *O-PD* open pancreaticoduodenectomy, *POD* postoperative day, *R-PD* robotic pancreaticoduodenectomy, *S-HA* subtle hypoattenuated area

### PSM analysis for comparing O-PD vs R-PD

**Fig. 2:**
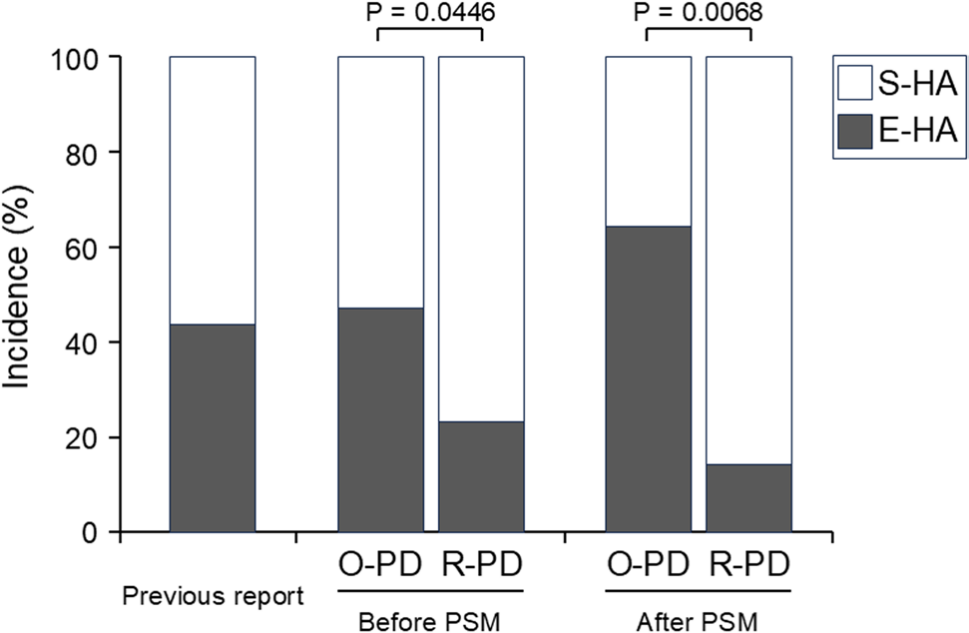
Distribution of patients exhibiting E-HA and S-HA, stratified by surgical approach. The distribution of patients exhibiting E-HA and S-HA is shown in patients who underwent O-PD or R-PD, both before and after PSM, and is compared with that in our previous report [17]. The incidence of E-HA in the R-PD group was significantly lower than in the O-PD group, both before and after PSM. E-HA, evident hypoattenuated area; O-PD, open pancreaticoduodenectomy; PSM, propensity-score matching; R-PD, robotic pancreaticoduodenectomy; S-HA, subtle hypoattenuated area

The above results still allowed the possibility of a significant association between the surgical approach and HA status. However, since some background factors of the two groups differed significantly, it remains unclear how the approach actually affects HA status, resulting in the incidence of CR-POPF. Therefore, PSM was performed to more fairly compare the R-PD and O-PD groups. The comparison after matching is shown in Table 5. In this comparison, the preoperative factors were comparable between the two groups. Given this comparable background, the drain amylase level on POD3 did not differ significantly between the two groups. On the other hand, the percentage of patients with E-HA was significantly lower in the R-PD group than in the O-PD group (14.3% vs 64.3%; *p* = 0.0068). The distribution of patients exhibiting E-HA and S-HA stratified by the surgical approach is summarized in Fig. 2. Furthermore, the R-PD group exhibited a significantly lower incidence of CR-POPF than did the O-PD group (14.3% vs 57.1%; *p* = 0.0180), while there was no significant difference in the incidence of the other complications (7.1% vs 7.1%; *p* > 0.9999).

**Table 5:** Clinical characteristics in patients in O-PD and R-PD groups after PSM

Factors	O-PD group (*n* = 14)	R-PD group (*n* = 14)	*P* value
Preoperative factors			
Age (years)	66 ± 10	66 ± 18	0.8789
Sex			0.4450
Male (%)	9 (64.3%)	7 (50.0%)	
Female (%)	5 (35.7%)	7 (50.0%)	
BMI (kg/m^2^)	23.2 ± 2.8	22.8 ± 2.8	0.6787
MPD diameter (mm)	2.9 ± 1.9	2.6 ± 1.5	0.6362
Pancreatic thickness (mm)	11.6 ± 2.6	12.4 ± 1.8	0.3254
Pancreatic texture			> 0.9999
Soft	14 (100%)	14 (100%)	
Hard	0 (0%)	0 (0%)	
Neoadjuvant therapy			> 0.9999
−	11 (78.6%)	11 (78.6%)	
+	3 (21.4%)	3 (21.4%)	
Tumor location			0.5647
Pancreas	10 (28.6%)	10 (16.7%)	
Bile duct	3 (66.7%)	4 (70.8%)	
Duodenum	1 (2.4%)	0 (8.3%)	
Others	0 (0%)	0 (4.2%)	
Intraoperative factors			
Operation time (min)	436 ± 90	588 ± 107	0.0004
Intraoperative blood loss (mL)	401 ± 273	195 ± 295	0.0663
Intraoperative transfusion			> 0.9999
−	14 (100%)	13 (92.9%)	
+	0 (0%)	1 (7.1%)	
Postoperative factors			
Drain amylase on POD 3 (U/L)	8145 ± 9146	5729 ± 6835	0.4357
HA			0.0068
S-EA	5 (35.7%)	12 (85.7%)	
E-HA	9 (64.3%)	2 (14.3%)	
Postoperative complication			
CR-POPF			0.0180
−	6 (42.9%)	12 (85.7%)	
+	8 (57.1%)	2 (14.3%)	
Other complications			> 0.9999
−	13 (92.9%)	13 (92.9%)	
+	1 (7.1%)	1 (7.1%)	

*BMI* body mass index, *CR-POPF* clinically relevant postoperative pancreatic fistula, *E-HA* evident hypoattenuated area, *MPD* main pancreatic duct, *O-PD* open pancreaticoduodenectomy, *POD* postoperative day, *PSM* propensity-score matching, *R-PD* robotic pancreaticoduodenectomy, *S-HA* subtle hypoattenuated area

. The results suggest that R-PD, in comparison with O-PD, led to a significantly lower percentage of patients with E-HA formation and subsequent incidence of CR-POPF.

## Discussion

Our previous study demonstrated the clinical impact of HA formation on predicting CR-POPF in the patients who received O-PD [17]. Building on this, here we aimed to verify this impact in a consecutive series of patients who received either O-PD or R-PD. The findings confirm that HA formation predicts CR-POPF, and further reveal that the incidence of E-HA formation was significantly lower in the R-PD group than in the O-PD group, suggesting an advantage of R-PD over O-PD in reducing HA formation and consequent CR-POPF.

**Fig. 3:**
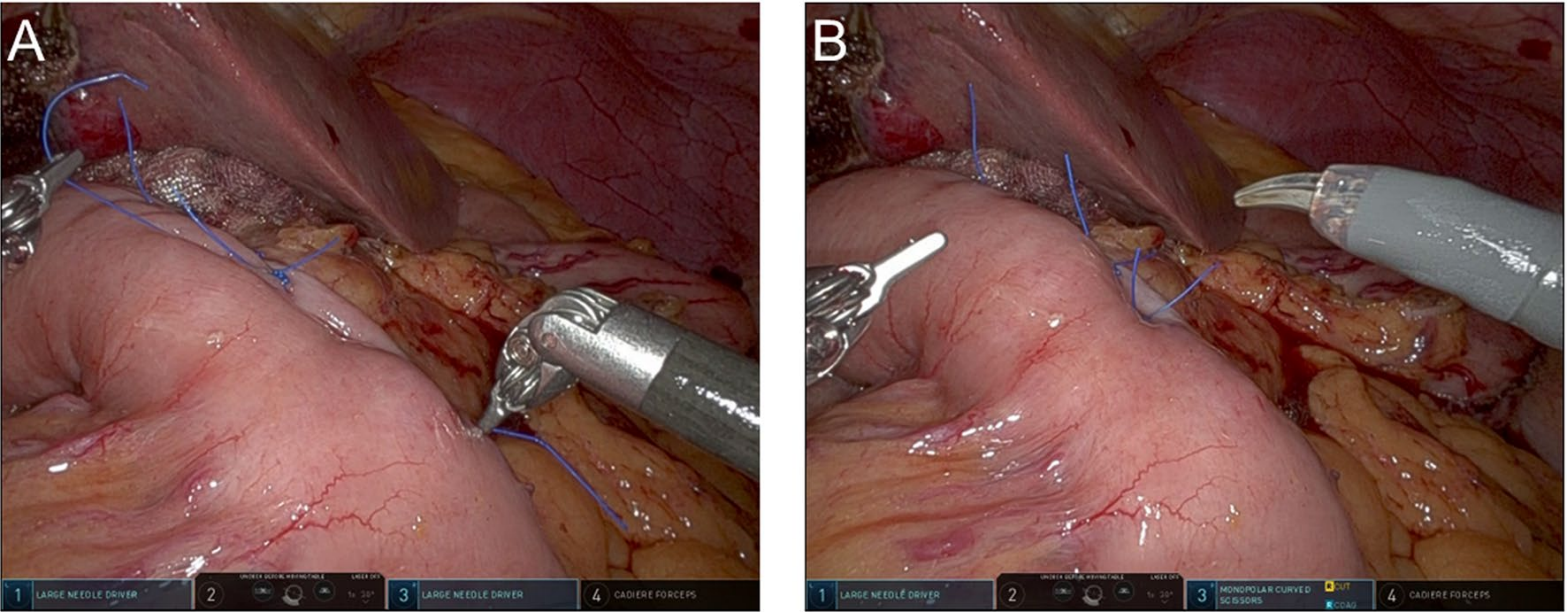
Photographs showing ligation on the jejunal serosa covering the pancreatic stump during the PJ procedure in R-PD. **A** The photograph shows the potentially looser ligation using 3–0 nonabsorbable monofilaments with the modified Blumgart method [18], which leads to less blood flow reduction at the PJ site in R-PD compared with O-PD. **B** The completed PJ anastomosis. O-PD, open pancreaticoduodenectomy; PJ, pancreatojejunostomy; R-PD, robotic pancreaticoduodenectomy

In our previous study, the analysis of the CT value of HA let us speculate that the HA area is not fluid collection or anastomosis separation, but rather pancreatic parenchyma with reduced blood flow. Taking the new results into account together with this speculation, we suggest that an unknown factor may have lessened the reduction of blood flow at the PJ site in patients who underwent R-PD in comparison with those who received O-PD. Unfortunately, we could not identify this factor. However, we initially speculated that the reduced blood flow might result from excessive tension when creating the PJ and/or over-mobilization of the remnant pancreas for the PJ anastomosis. Since the PJ procedure is the same in both O-PD and R-PD, including the mobilization of the remnant pancreas, the factor may lie in the differences in the strength of ligation on the jejunal serosa covering the pancreatic stump during the PJ procedure. In robotic surgery, the lack of tactile sensation might lead to looser ligations due to concerns about applying excessive force, which could cause the thread to break. This may lead to less blood flow reduction at the PJ site and, consequently, a lower incidence of HA formation in R-PD [21, 22]. The photographs in Fig. 3 show the potentially looser ligation on the jejunal serosa covering the pancreatic stump during the PJ procedure. To test this hypothesis, we plan to analyze blood flow at the PJ site in the near future.

When considering the clinical applications of these results, one is that the progression to CR-POPF can be predicted by the HA findings in the patients exhibiting BL POPF, regardless of whether they underwent O-PD or R-PD. In this case series, the presence of an E-HA predicted progression to CR-POPF with 89.4% sensitivity and 85.1% specificity. When stratified by the surgical approach, these values were, respectively, 100% and 76.0% in O-PD, and 75.0% and 95.5% in R-PD, indicating the potential clinical utility of HA findings in both groups. Another important point concerns the incidence of E-HA formation based on the surgical approach. As summarized in Fig. 2, the incidence of E-HA in the O-PD group was consistent with our previous report [17]. The incidence was significantly lower in the R-PD group than in the O-PD group before PSM, and this trend was confirmed after PSM. Although the advantage of R-PD over O-PD remains inconclusive, our retrospective study provides valuable evidence suggesting that R-PD may have an edge over O-PD [6–10, 12]. Finally, as mentioned earlier, we have speculated on the underlying mechanism of HA formation, especially considering the reduced percentage of E-HA in the patients with R-PD. Understanding this mechanism, which leads to CR-POPF, could offer insights for preventing CR-POPF not only in R-DP but also in O-PD.

This study has several limitations. First, although the impact of HA was also confirmed in patients who underwent R-PD, the study is retrospective and includes a small number of patients, necessitating caution in interpreting the results. In particular, the small sample size applies to both pre- and post-PSM analyses, making it a significant limitation. Actually, we had considered collecting additional cases prior to reporting the results of the present study, but we prioritized disseminating the current findings and chose to publish with the currently available sample size. In the future, we plan to increase the number of cases and conduct further validation in conjunction with the aforementioned blood flow evaluation. Furthermore, due to the retrospective study design, R-PD and O-PD were not randomly assigned to the included patients, indicating that they remained incomparable even after performing PSM for the comparison. This represents a limitation inherent to the study design. Second, the included patients in this study were limited to those who met the diagnostic criterion of BL and underwent CE-CT assessment on PODs 3–14. In addition, regarding the timing of CT, although it was within the range of 3 to 14 days, it was not strictly set to a specific day. Therefore, while there was no significant difference in the duration from the surgery to CT between the E-HA group and the S-HA group, the results of this study may include potential bias related to the timing of CT. This limitation means that the findings in this study may apply only to similar patients. If applied otherwise, the universality of the results of this study might be compromised.

In summary, this study confirmed the clinical impact of HA formation in predicting CR-POPF in patients who received PD, including both O-PD and R-PD. Furthermore, the results suggest that R-PD, compared with O-PD, significantly reduces the incidence of E-HA formation, indicating a potential advantage of R-PD over O-PD.
